# Newly diagnosed isolated myeloid sarcoma–paired NGS panel analysis of extramedullary tumor and bone marrow

**DOI:** 10.1007/s00277-020-04313-x

**Published:** 2020-10-27

**Authors:** Nils W. Engel, Jochim Reinert, Nora M. Borchert, Victoria Panagiota, Razif Gabdoulline, Felicitas Thol, Michael Heuser, Walter Fiedler

**Affiliations:** 1 Department of Oncology, Haematology and Bone Marrow Transplantation with Section Pneumology, Hubertus Wald Tumorzentrum, University Medical Center Hamburg-Eppendorf, Hamburg, Germany; 2 Department of Hematology, Hemostasis, Oncology, and Stem Cell Transplantation, Hannover Medical School, Hannover, Germany

**Keywords:** Myeloid sarcoma, CHIP, Clonal evolution, NGS sequencing, Homing

## Abstract

Isolated myeloid sarcoma (MS) is a rare malignancy in which myeloid blast forms tumors at various locations while the bone marrow (BM) remains cytomorphologically free from disease. We analyzed isolated MS from four patients and their BMs at initial diagnosis and follow-up, using a custom next-generation sequencing (NGS) panel. We observed possible clonal evolution and a clonal hematopoiesis of indeterminate potential (CHIP)-like finding in the BM of one of three cases with detectable mutations. Clinical presentation of one patient suggested extramedullary confined homing of blasts to distal sites in the relapse situation still sparing the BM. In summary, our findings shall motivate future work regarding signals of extramedullary blast trafficking and clonal evolution in MS.

**Supplementary Information:**

The online version contains supplementary material available at 10.1007/s00277-020-04313-x.

## Introduction

Myeloid sarcoma (MS) is characterized as an extramedullary tumor composed of myeloid blasts. It may manifest simultaneously as a part of acute myeloid leukemia (AML), as a progression of myeloproliferative neoplasms or myelodysplastic syndromes, or it may arise at relapse, especially in patients following allogenic hematopoietic stem cell transplantation [1]. De novo isolated MS without bone marrow (BM) infiltration is a rare variant of MS, but is usually, although not always [2], a harbinger of subsequent BM blast infiltration and overt AML with a short delay [3].

Isolated MS was historically defined on a cytological or histological level, and one may wonder whether this concept can persist in the time of modern sensitive PCR-based detection methods of minimal residual disease (MRD) [4]. Also, a growing understanding of clonal heterogeneity and evolution in healthy and neoplastic hematopoiesis, such as clonal hematopoiesis of indeterminate potential (CHIP) [5], demands a NGS-based parallel assessment of MS specimen and BM samples in isolated MS cases in order to explore any manifestation of those phenomena in this unique scenario.

## Materials and methods

We identified four cases of de novo isolated MS, diagnosed between 2015 and 2017 at our institution suitable for subsequent assessment. For all cases, material for DNA isolation from MS primary sites (formalin-fixed paraffin-embedded (FFPE) tissue) and BM (fresh aspirate or FFPE biopsy samples) at initial diagnosis was available. For two of these patients, follow-up samples (blood or BM aspirate) were available during complete response, and in one patient paired samples from a distant extramedullary site and BM at relapse. DNA was isolated according to standard protocols. Samples at initial diagnosis and relapse were submitted to a custom TruSight myeloid NGS sequencing panel approach covering 46 entire genes or hotspots associated with myeloid leukemias (Supplementary Table S1), as previously described [6]. Identified variants were validated using a sensitive error-corrected amplicon-sequencing approach with a sensitivity threshold of 0.015%, as previously described [7]. Amplicon-sequencing was also applied to follow-up samples in CR in order to trace for potential residual clones.

## Results and discussion

### Clinical case descriptions

Patient 1 was a 71-year-old Caucasian male, who was diagnosed with isolated MS in February 2015. PET-CT scan showed an occipital cutaneous tumor with adjacent bone lesions of the left parietal calvaria. Diagnosis of MS was established from an initial attempt of surgical tumor resection. Histopathological examination of BM biopsy showed no evidence of AML. Therapy included two courses “7 + 3” induction and one course of intermediate dose Ara C (IDAC) consolidation (cytarabine 1000 mg/qm every 12 h for 3 days), followed by tomotherapy of the calvaria (50 Gy fractionated). PET-CT scan in October 2015 after completion of therapy was interpreted as PET-negative CR. The patient remained in remission until last follow-up, 54 months after diagnosis.

Patient 2 was a 30-year-old male of Arab descent, who was diagnosed with isolated MS in August 2015. PET-CT scan revealed multifocal bilateral enlarged lymph nodes at cervical, axillar, iliac, and inguinal sites, and definite diagnosis was established from histopathological evaluation of a left submandibular lymph node. BM was unaffected by cytology, flow cytometry and histomorphology. The patient received standard AML induction therapy with “7 + 3” chemotherapy and achieved PET-negative complete response after the second course. The patient was scheduled for allogenic matched unrelated donor stem cell transplantation (HSCT) (10/10 HLA match) after myeloablative conditioning with TBI-CY (12 Gy fractionated + cyclophosphamide 120 mg/qm + ATG 60 mg/kg). Twenty-six days after transplantation and 9 days after leukocyte engraftment (≥ 1 × 10^9^/l), the patient died as a consequence of septic shock with multiorgan failure.

Patient 3 was a 79-year-old Caucasian male, who was diagnosed with isolated MS in May 2016. PET-CT scan showed a single subcutaneous soft tissue mass with pathological glucose uptake at the left posterior upper arm and diagnosis was histopathologically established from biopsy. Cytomorphology and flow cytometry of BM were normal. Therapy was initiated with two courses of 7 + 3 induction therapy followed by two courses of IDAC. A local radiotherapy (30 Gy fractionated) was added. PET-CT scan in January 2017, 5 weeks after completion of therapy, showed a persisting tumor mass at the original site with less intense, but still suspicious glucose uptake. Consecutive follow-up monitoring was conducted with whole-body MRI scans and the former MS lesion completely resolved by October 2017 on MRI. Last follow-up in our clinic was consistent with sustained remission 17 months after diagnosis.

Patient 4 was a 39-year-old Caucasian male, who was diagnosed with isolated MS in December 2017. According to PET-CT, MS manifested as a single bone lesion of the left ilium involving its acetabular portion. The patient’s BM was unaffected on cytological and flow cytometric assessment. Treatment was conducted with two courses of “7 + 3” induction therapy and three courses of IDAC consolidation, followed by local radiotherapy (50 Gy fractionated). PET-CT scan in September 2018 after completion of therapy showed only slightly enhanced glucose uptake at the initially affected site, which was interpreted as likely post-radiogenic. However, 19 months after diagnosis in July 2019, the patient was admitted again with suspected disease relapse with multifocal bone lesions and a left inguinal lymph node with pathological glucose uptake on PET-CT scan. A localized lesion in the left hepatic lobe, visible on sonography and MRI, underwent biopsy for histopathological confirmation of MS relapse. Still, there was no evidence for AML infiltration of BM on cytological and flow cytometric assessment. Thus, this patient’s disease presentation of isolated MS at initial diagnosis was preserved in the situation of relapse. The patient received one course of FLAG-IDA and was scheduled for allogenic 9/10 HLA mismatched sibling donor HSCT after additional induction with one course of FLAMSA and myeloablative conditioning with TBI-FLU-post-CY (12 Gy fractionated + fludarabine 60 mg/qm + cyclophosphamide 100 mg/kg post-transplantation) in September 2019. PET-CT scan 55 days after HSCT displayed normal global glucose metabolism, interpreted as PET-negative CR. The patient was in remission at last follow-up 31 months after diagnosis and 10 months after HSCT.

### Molecular findings from NGS panel sequencing

NGS panel sequencing of DNA isolated from isolated MS specimen of these four patients at initial diagnosis resulted in identification of molecular aberrations in three out of four cases (Table 1; patients 1, 2, and 4) and no mutation in the remaining patient. However, parallel assessment of the cytomorphological unaffected BM did not detect these variants in two out of three cases (patients 1 and 4) by means of sensitive amplicon sequencing. Thus, BM involvement was not detectable on the molecular level in these patients, and especially, no presence of a precursor CHIP lesion could be detected, suggesting that malignant transformation occurred at the extramedullary MS site rather than within the BM.

**Table 1:** Clinical and molecular characteristics of four patients with de novo isolated myeloid sarcoma

Case	Sex/age	Site	History	Gene	Variant	Initial diagnosis		1. CR Follow-up	Relapse		Therapy	Subsequent clinical history
						Tumor VAF (%)	BM VAF (%)	VAF (%)	Tumor VAF (%)	BM VAF (%)		
1	M/71 years	Cutaneous lesion, occipital	de novo isolated MS	*DNMT3A*	c.1180G>A (p.Asp394Asn)	77.4	Not detectable	Not detectable^*^	NA		2x DA (7+3). 1x IDAC. Tomotherapy (50 Gy)	Sustained remission at last follow-up, 54 months after diagnosis.
				*DNMT3A*	c.880G>A (p.Glu294Lys)	14.43	Not detectable	Not detectable^*^				
				*TET2*	c.3973C>T (p.His1325Tyr)	11.32	Not detectable	Not detectable^*^				
				*TP53*	c.1006G>A (p.Glu336Lys)	42.7	Not detectable	Not detectable^*^				
2	M/30 years	Multifocal lymph nodes (bilaterally cervical, axillar, iliac and inguinal)	de novo isolated MS	*ETV6*	c.1187_1188insCGCTACGGACC (p.Arg396SerfsTer13)	21.9	1.42	NA	NA		2x DA (7+3). Allogenic matched unrelated donor HSCT (10/10 HLA match) after myeloablative conditioning with TBI-CY.	PET-negative CR after induction. Dead due to septic shock 3 months after diagnosis/26 days after HSCT.
				*STAG1*	c.151C>T (p.Arg51Ter)	22.9	Not detectable					
3	M/79 years	Subcutaneous lesion, left posterior upper arm	de novo isolated MS	no variants detectable		NA		NA	NA		2x DA (7+3). 2x IDAC. Radiotherapy (30 Gy).	Sustained remission at last follow-up, 17 months after diagnosis.
4	M/39 years	Bone lesion left ilium involving acetabular portion	de novo isolated MS	*FLT3* (atypical variant)	c.1834T>C (p.Phe612Leu)	16.6	Not detectable	Not detectable^#^	46.4^†^	Not detectable	*Initially:* 2x DA (7+3). 3x IDAC. Radiotherapy (50 Gy). *At time of relapse:* 1x FLAG-IDA. Allogenic sibling donor HSCT (9/10 HLA mismatch) after myeloablative conditioning with FLAMSA + TBI-FLU-post-CY.	PET-negative CR after induction. Disease relapse 19 months after diagnosis (isolated MS with liver and multiple bone lesions). 2. PET-negative CR 55 days after HSCT. Sustained remission at last follow-up, 31 months after diagnosis.
				*FLT3* (atypical variant)	c.1765T>C (p.Tyr589His)	9.9	Not detectable	Not detectable^#^	28.9^†^	Not detectable		
				*MYC*	c.218C>A (p.Thr73Asn)	68.6	Not detectable	Not detectable^#^	45^†^	Not detectable		
				*NPM1*	c.863_864insCATG (p.Trp288CysfsTer12)	41.2	Not detectable	Not detectable^#^	39.1^†^	Not detectable		
				*PHF6*	c.905A>G (p.His302Arg)	70.9	Not detectable	Not detectable^#^	96.7^†^	Not detectable		

*M* male, *MS* myeloid sarcoma, *VAF* variant allele frequency, *BM* bone marrow, *HSCT* hematopoietic stem cell transplantation, *CR* complete remission, *DA (7 + 3)* daunorubicin 60 mg/qm days 3–5 + cytarabine 100 mg/qm days 1–7 as continuous infusion, *IDAC* intermediate dosed cytarabine 1000 mg/qm twice daily days 1, 3, and 5, *TBI-CY* 12 Gy fractionated + cyclophosphamide 120 mg/qm + ATG 60 mg/kg, *FLAG-IDA* fludarabine 30 mg/qm, cytarabine 2000 mg/qm, each day 1–5, idarubicin 10 mg/qm day 1–3, G-CSF 5 μg/kg from day 6, *FLAMSA* fludarabine 30 mg/qm, amsacrine 100 mg/qm, cytarabine 2000 mg/qm, each day 1–4, *TBI-FLU-post-CY* 12 Gy fractionated + fludarabine 60 mg/qm + cyclophosphamide 100 mg/kg post-transplantation, *NA* not applicable

^a^Blood

^b^Bone marrow

^c^Liver biopsy

Patient 1’s MS showed typical mutations described for CHIP, namely *DNMTA3* and *TET2* [8]. As we did not detect these variants in the corresponding BM, this may foster the hypothesis that CHIP may also occur extramedullary and be confined locally. For the first *DNMT3A* variant (variant allele frequency (VAF) 77.4%), an additional *TP53* mutation in a subclone (VAF 42.7%) may represent clonal evolution locally in this case, resulting in malignant transformation and clinically overt disease.

In patient 2, an *ETV6* mutation was detected in BM and MS. Since the *ETV6* mutation in the BM sample had a VAF of only 1.42%, which was much higher in the extramedullary tumor (21.9%), it may represent true CHIP. The original definition of CHIP set a VAF cutoff of ≥ 2%, but this limit took into account methodological limitations and was arbitrary [8].

Clonal hematopoiesis (CH) defined by a variant of *ETV6* is uncommon compared with variants frequently observed in CHIP [8].

In previous studies, several other uncommon variants for CH have been reported in unaffected BM specimen of isolated MS cases (*IDH2* [9], *NFE2*, *ODF1*, *TRAFD1* [10], *RUNX1*, *GATA2*, *FLT3*, and *NPM1* [11], suggesting a unique pattern of clonal evolution and pathogenesis of isolated MS. The mentioned studies reported simultaneous NGS-based parallel assessment of MS sites and BM for five, two, and six cases of de novo isolated MS, respectively, and detected evidence for CH in unaffected BM in one (20%), two (100%), and three (50%) of analyzed cases. In our cohort, only one of three patients with detectable mutations and isolated de novo MS (patient 2) had evidence of CHIP in the BM at presentation.

In patient 2’s MS, an additional *STAG1* mutation outside the BM was found. This indicates that clonal evolution had occurred at an extramedullary site and that this additional mutation may have resulted in the appearance of an overt clinical manifestation.

After initial therapy with induction chemotherapy and radiation of the solitary extramedullary site, previous variants remained undetectable by amplicon sequencing in blood or BM of patients 1 and 4 in continuous CR.

Patient 4 had a multifocal extramedullary relapse, again without BM involvement. The same mutational profile was found in the distant relapse site compared with initial manifestation. Interestingly, the sites of exclusively extramedullary blast infiltration suggested by PET-CT at relapse (lumbar vertebrae 2 and 4, left inguinal lymph node) and by sonography, confirmed by biopsy (liver), differed from the affected site at initial diagnosis (left ilium). The later showed no significant glucose uptake by PET-CT at relapse and was likely unaffected at this time point. Together, these findings suggested extramedullary homing of myeloid blasts in distant sites at relapse without BM involvement. Alternate homing appears to be the hallmark difference between MS and AML, suggesting an aberrant homing signal between these entities [12]. The documented disease presentation of patient 4 raises the question whether there might be even distinct homing signals for trafficking of myeloid blasts between different extramedullary compartments—a question worth to explore in future studies.

### Conclusion and future prospect

In some cases, isolated MS might arise exclusively extramedullary without precursor cell populations in the BM (as suggested by molecular results from patients 1 and 4, Table 1).

Alternatively, BM precursor clones of isolated MS might be defined by yet uninvestigated features (e.g., uncommon AML/CHIP-associated mutations not captured by our NGS panel, cytogenetic aberrations). Recently, an uncommon accumulation of *NFE2* mutations in isolated MS patients (4/6; 67%) has been carved out via whole-exome sequencing [10], also detectable in one of two unaffected BM specimen. Experimental evidence has indeed established *NFE2* aberrations as leukemogenic, MS promoting drivers in a murine model [13].

CH has been mainly assessed on the level of gene mutations, but might be identifiable on the level of chromosomal alterations as well with future methodological improvements. Adding systematic parallel characterization of chromosomal aberrations in isolated MS samples and suspected pre-leukemic BM clones will represent a special challenge for future investigations, given the level of sensitivity needed to reliably elucidate different subclones via cytogenetic information [14] as well as the need to gather such information from FFPE MS samples, the latter of which is already possible [15–17].

## Electronic supplementary material

ESM 1 (DOCX 14 kb)

## Data Availability

Raw sequencing datasets analyzed in this study can be made available on reasonable request.
