# Supplementary material for: Newly diagnosed isolated myeloid sarcoma–paired NGS panel analysis of extramedullary tumor and bone marrow
Source: Ann Hematol. 2020 Oct 27;100(2):499–503. doi: 10.1007/s00277-020-04313-x (PMC7817572; doi:10.1007/s00277-020-04313-x)
Supplement: Supplementary file 1 — (DOCX 14 kb) [file 277_2020_4313_MOESM1_ESM.docx]

**Table S1.** Genes covered by our custom myeloid panel (based on GRCh37/hg19).

| **Gene** | **Exons** | **Gene** | **Exons** | **Gene** | **Exons** |
| --- | --- | --- | --- | --- | --- |
| ASXL1 | 12 | GATA2 | 2-6 | RUNX1 | complete |
| ASXL2 | 11+12 | IDH1 | 4 | SETBP1 | 4 |
| BCOR | complete | IDH2 | 4 | SF3B1 | 13-16 |
| BCORL1 | complete | JAK2 | 12, 14 | SMC1A | 2, 11, 16, 17 |
| BRAF | Exon15 | KDM6A | complete | SMC3 | 10, 13, 19, 23, 25, 28 |
| CALR | 9 | KIT | 2, 8-11, 13, 17 | SRSF2 | 1 |
| CBL | 8, 9 | KRAS | 2-5 | STAG1 | complete |
| CEBPA | complete | MPL | 10 | STAG2 | complete |
| CSF3R | 14-17 | MYC | 2 | TET2 | 3-11 |
| CSNK1A1 | 3, 4 | NF1 | complete | TP53 | 2-11 |
| DDX41 | complete | NPM1 | 11 | U2AF1 | 2, 6 |
| DNMT3A | complete | NRAS | 2-5 | WT1 | 7, 9 |
| ETNK1 | 3 | PHF6 | complete | ZBTB7A | 2, 3 |
| ETV6 | complete | PPM1D | 1-6 | ZRSR2 | complete |
| EZH2 | complete | PTPN11 | 3, 13 |  |  |
| FLT3 | 14-16, 20 | RAD21 | complete |  |  |
